# Mouse model of specific fragrance allergy: the haptenous state determines site-specific tolerance or allergy

**DOI:** 10.1186/2045-7022-4-S2-P32

**Published:** 2014-03-17

**Authors:** Franziska Roth-Walter, Anna Moskovskich, Anna Willendorfer, Cristina Gomez-Casado, Daniela Quiroga Pollak-Monje, Erika Jensen-Jarolim

**Affiliations:** 1 University of Veterinary Medicine, Messerli Research Institute, Comparative Medicine, Vienna, Austria; 2 Technical University Madrid, Center for Plant Biotechnology and Genomic, Madrid, Spain; 3 Medical University of Vienna, Department of Neurophysiology & Neuropharmacology, Vienna, Austria; 4 Medical University of Vienna, Department of Pathophysiology and Allergy Research, Vienna, Austria

## Background

Cinnamon aldehyde (CA) represents a ubiquitous fragrance being respired, contacted by skin or consumed via the oral route and it may lead to type I. and IV. hypersensitivity. Here, we sought to investigate whether the encounter of CA as a hapten or coupled to a carrier influences whether the organism responds allergic or tolerant.

## Methods

Cinnamaldehyde (CA) alone, or conjugated with keyhole limpet hemocyanin (CA-KLH) was applied nasally six times on two consecutive days in biweekly intervals. Subsequently, ears were painted with cinnamaldehyde and ear swelling measured after 24 and 48h. Mice were also challenged by oral gavage of 2mg CA and body temperature was measured for 30min. Cytokine profile of stimulated splenocytes as well as CA-specific antibodies and corticosteron-levels were assessed by ELISA.

## Results

Only in mice sensitized with CA-KLH a specific immune response with CA-specific IgG1, IgE, IgG2a and IgA levels as well as elevated IFNγ could be observed. CA-KLH sensitized mice also showed significant ear swelling response to CA after 24h. However, CA-KLH mice were protected from anaphylaxis upon oral challenge. In contrast, although no specific antibody and cytokine response could be detected in mice sensitized with the hapten CA only, 40% reacted upon oral challenge with CA by an anaphylactic decrease in body temperature and decreased physical activity and. In contrast, they were unresponsive upon epicutaneous challenge with CA. Interestingly, corticosteron levels of CA-sensitized mice were the lowest between the groups.

## Conclusions

We conclude that fragrances may via the nasal route indeed lead to specific type I hypersensitivity, but that the type and extent of response is critically determined by their antigenic state - hapten or complete antigen.

